# Pathological Features and Surgical Strategies of Cervical Deformity

**DOI:** 10.1155/2020/4290597

**Published:** 2020-05-12

**Authors:** Xiangyao Sun, Siyuan Sun, Chao Kong, Wei Wang, Tongtong Zhang, Junzhe Ding, Xiangyu Li, Shibao Lu

**Affiliations:** ^1^Department of Orthopedics, Xuanwu Hospital Capital Medical University, China; ^2^National Clinical Research Center for Geriatric Diseases, China; ^3^Department of Interdisciplinary Life Science, Purdue University, West Lafayette, IN 47907, USA; ^4^Department of Orthopedics, ChuiYangLiu Hospital Affiliated to Tsinghua University, China

## Abstract

Cervical deformity (CD) is a kind of disorder influencing cervical alignment. Although the incidence of CD is not high, this deformity can cause not only pain but also difficulties in daily activities such as swallowing and maintaining upright position. Even though the common cause of cervical deformity is still controversial, previous studies divided CD into congenital deformity and secondary deformity; secondary deformity includes iatrogenic and noniatrogenic deformity according to pathogenic factors. Due to the lack of relevant studies, a standardized evaluation for CD is absent. Even though the assessment of preoperative condition and surgical planning mainly rely on personal experience, the evaluation methods could still be summarized from previous studies. The objective in this article is to summarize studies on cervical scoliosis, identify clinical problems, and provide directions for researchers interested in delving deep into this specific topic. In this review, we found that the lack of standard classification system could lead to an absence of clinical guidance; in addition, the osseous landmarks and vascular distributions could be variable in CD patients, which might cause the risk of vascular or neurological complications; furthermore, multiple deformities were usually presented in CD patients, which might cause chain reaction after the correction of CD; this would prevent surgeons from choosing realignment surgery that is effective but risky.

## 1. Introduction

Cervical deformity (CD) is always defined as a kind of disorder influencing cervical alignment. It is commonly associated with increased tension and anterior pressure in the spinal cord, and then, myelopathy occurs; a patient can be diagnosed with CD if his Cobb angle of the cervical spine is over 10°, which is similar to thoracic and lumbar scoliosis [1]. Although the incidence of CD is not high, this deformity can cause not only pain but also difficulties in daily activities such as swallowing and maintaining upright position [2]. Considering complicated etiology, highly variable clinical manifestation, and risky operations, the assessment and treatment of CD remain a complicated problem for spine surgeons. However, in contrast to thoracolumbar scoliosis, spine surgeons have limited understanding of CD. In addition, because CD is rare, most researches are presented as case reports of case series. To our knowledge, no literature has systematically reviewed the pathological features and treatment of CD. Therefore, the objective in this article is to summarize studies on CD, identify clinical problems, and provide directions for researchers interested in delving deep into this specific topic.

## 2. Pathological Features

Even though the common cause of cervical deformity is still controversial, previous studies divided CD into congenital deformity and secondary deformity; secondary deformity includes iatrogenic and noniatrogenic deformity according to pathogenic factors (Table 1) [3–8]. Understanding the pathological features of CD can do good to the selection of therapeutic interventions.

### 2.1. Congenital Deformity

Congenital deformity is identified as cervical malalignment in coronal plane caused by abnormal vertebral development. Failure in abnormal vertebral development can create a lateral curvature of the spine and form a deformity [7]. The severity of congenital scoliosis is determined by type and region of the deformity. Sagittal malalignment is commonly seen in CD, which is usually combined with kyphotic or lordotic deformity; in some cases, anomalies of other systems, including the cardiovascular system or urogenital system, could also be detected [8]. Only cases with genetic syndromes have certain causes; others are sporadic without determined etiologies.

#### 2.1.1. Klippel-Feil Syndrome

Klippel-Feil syndrome (KFS) is one of the most common causes of congenital CD. Previous studies reported the incidence of CD in KFS which was 70.2%-78.2% [9, 10]. Maurice Klippel and Andre Feil first reported KFS in 1912; the true cause of KFS has been undetermined ever since [11]. Despite the individual symptoms which are variable, congenital fusion of 2 or more cervical vertebrae is the most unique characteristic of KFS; in addition, over 50% of KFS patients presented with a classic triad: a short neck, limited neck motion, and a low posterior hairline [12]. Samartzis et al. [10] stated that the most commonly fused segments are C5-6 and C2-3 with a prevalence of CS to occur in 53.3% of KFS patients. The most commonly used radiology-based classification system was reported by Samartzis et al. [13]: type I, single congenitally fused cervical segment; type II, multiple noncontiguous, congenitally fused segments; and type III, multiple contiguous, congenitally fused cervical segments. This classification describes KFS in detail and is of great significance for clinical evaluation of KFS. In addition, they found that there was a linear relationship between prevalence of CD and KFS classification; in their study [10], CD was rarely seen in type I KFS; the incidences of CD were 60% and 70% in type II and type III patients, respectively. Gruber et al. [14] found that none of the type I patients exhibit CD, which proves the correctness of Samartzis classification. Therefore, Samartzis classification system may be able to help predict the existence of CS. However, further studies are needed to validate this relationship.

Most of the previous studies stated that the incidence of CD was high in KFS patients; however, most of them did not discuss cervical or neurological symptoms [9, 10, 12]. Some studies concluded that most young KFS patients were asymptomatic, while adult patients, due to progressive cervical fusion, might present degenerative manifestations [15]. In contrast, other studies found that up to 50% of young patients also show cervical spine-related symptoms [12, 16]. Theiss et al. [17] reported that none of their KFS patients have cervical-related symptoms initially; after 10 years, although with dramatic radiology appearances, only 22% patients had cervical or cervical-related symptoms, 2 of which require surgery; in addition, they concluded that KFS patients with congenital stenosis or fused to the cervicothoracic junction are at greater risk of gaining corresponding symptoms.

#### 2.1.2. Hemivertebrae

Hemivertebrae is a congenital deformity caused by failure of formation and fusion in one side of the vertebral body with an incidence of 0.01% in newborns, which is much rarer than thoracic and lumbar hemivertebrae [18]. Since Deburge and Briard and Winter and House first reported cervical hemivertebrae in 1981, cervical hemivertebrae have only been reported in case reports or case series [19, 20]. Xue et al. [21] pointed out that 14 out of 28 KFS patients in their research had cervical hemivertebrae, which indicated that cervical hemivertebrae tend to be a part of congenital deformity, instead of represented as an individual deformity. Because there is only one functional disc in a functional cervical segment with hemivertebrae, activity of the affected segment can be severely limited; progressive tilt above the hemivertebrae can be frequently observed with a progression rate of 1° to 3.5° annually, which is usually combined with compensatory curves below the hemivertebrae [22]. Considering conservative treatment cannot halt its relentless progression, early surgical intervention should be considered in young cervical hemivertebra patients [21].

#### 2.1.3. Goldenhar Syndrome

Goldenhar syndrome, also known as oculoauricular vertebral (OAV) syndrome, is a rare kind of CD; it is characterized by failed development of the nose, ear, lip, soft palate, mandible, and first and second brachial arches; its common clinical manifestations include craniofacial microsomia, ocular dermoid cysts, vertebral anomalies, and cardiac or renal defects [23]. Although most of Goldenhar patients were sporadic, a previous study reported that there was an inheritance tendency in this disease [24]. Vertebral deformities in Goldenhar patients include hemivertebrae, vertebral fusion, scoliosis, spine bifida, and occipitalization of the atlas [25]. It has been reported that most of these patients did not need surgical treatment [6]. The prevalence of CD might be underestimated in Goldenhar patients, considering not all CD would result in clinically obvious deformity [23]. Thus, more studies are required to investigate the relationship between CD and Goldenhar syndrome. Early diagnosis and early treatment should be carried out for Goldenhar patients. Asymptomatic patients should be followed up every 6 months; patients with severe CD that might cause neurological symptoms need neurological evaluation every 3-6 months; extension-flexion plain radiography is very important in the follow-up [23].

#### 2.1.4. Neurofibromatosis Type 1

Neurofibromatosis is an autosomal dominant hereditary disorder, which is characterized by abnormal proliferation of neural crest cell. Anomalies can occur in both the peripheral nervous system and central nervous system. Neurofibromatosis includes two types: neurofibromatosis type 1 (peripheral neurofibromatosis or von Recklinghausen's disease) and neurofibromatosis type 2. Neurofibromatosis type 1 (NF-1), the incidence of which is 0.33%, is more common than neurofibromatosis type 2 (NF-2); NF-1 are frequently associated with musculoskeletal system defect, especially deformity; the incidence is 10%-64% [26]. Deformity in NF-1 can be divided into dystrophic deformity and nondystrophic deformity according to natural history and characteristics [27]. Dystrophic deformity occurs earlier than nondystrophic deformity; in addition, dystrophic deformity has worse prognosis compared with nondystrophic deformity. CD in NF-1, which is characterized by low prevalence and low symptomatic rate, attracts fewer attentions than thoracic or lumbar deformity. It was reported that CD in NF-1 was commonly associated with dysplastic deformity of the thoracic spine; in addition, symptomatic NF-1 patients with CD were more likely to suffer from cervical stenosis and nerve root compression, which would require surgical intervention [28].

### 2.2. Iatrogenic CD

Operational errors in preoperative position, size of instrument, amount of depression, location and size of bone graft, and use of postoperative immobilization can both cause malalignment of the cervical spine. Even the most experienced surgeon may accidentally create an iatrogenic CD. In order to avoid iatrogenic deformity, surgeons need to pay extra attention and maintain a high level of awareness during surgery [5]. One factor associated with iatrogenic cervical malalignment is the exposure of disc space during surgery. Failure to expose the lateral margin of vertebral body to reach the extension of transverse process will increase the likelihood of performing an asymmetric discectomy or corpectomy; in addition, this can cause the malposition of bone graft or implants [5, 29]. Considering uncovertebral joints are reliable osseous landmarks, the exposure of these structures can contribute to performing symmetric discectomy or corpectomy during surgery [6].

Iatrogenic CD can also be caused by instrumentations and bone grafts. If bone grafts were not enough to fill the disk space in discectomy surgery, intervertebral disc space would collapse; this could cause CD [8]. Therefore, grafts should be placed as many as possible in the disc space. Instrumentations are widely used in cervical corpectomy. It was reported that oversized implants would cause asymmetric corpectomy, which might result in postoperative CD [7]. In addition, although careful use of anterior cervical plate could prevent cervical kyphosis, an oversized cervical plate would cause plate-induced deformity [5]. If the screw accidentally penetrated the adjacent disc space, this disc space could degenerate and collapse rapidly, and then, CD would occur [30].

Another factor associated with iatrogenic CD is the preoperative position. Both shoulders of a patient are usually preoperatively taped to gain a better visualization of the spine; however, iatrogenic CD may occur if one side of the shoulder is taped below the other side; therefore, another surgeon should observe and keep both shoulders level [31]. If iatrogenic CD unfortunately occurred in one functional segment, the adjacent segments would compensate and few symptoms could be noticed; if iatrogenic CD occurred after a multilevel surgery, clinical cervical imbalance would happen [5, 6].

Age is also an important factor associated with iatrogenic CD. Young patients should be particularly concerned. The development of unmatured cervical spine can be easily interrupted by surgery or radiotherapy; in addition, resection of a tumor in the cervical spine or radiotherapy may destroy the ossification center on one side of the cervical spine, which will cause cervical malalignment a few years later [22]; unexpected postoperative scoliosis may also occur in adult CD patients. In patients with severe CD, their spines will manage to rebalance themselves so that their head can remain in a neutral position; if their CDs are corrected, the compensatory spines may tilt their head to the opposite side [5].

### 2.3. Noniatrogenic Secondary CD

The incidence of noniatrogenic secondary CD is much lower than that of other kinds of CD. Previous studies reported that etiology included tumor, trauma, or rheumatoid arthritis (RA), both of which could cause destruction of the vertebral body with or without subsequent vertebral fusion [8]. Under these circumstances, the activity of the destructed segment would be limited; then, other segments above or below this segment would compensate for this situation [6]. Therefore, timely surgical treatment of noniatrogenic secondary CD is very important.

## 3. Clinical Assessment

Due to the lack of relevant studies, a standardized evaluation for CD is absent. Even though the assessment of preoperative condition and surgical planning mainly rely on personal experience, the evaluation methods could still be summarized from previous studies.

### 3.1. Medical History and Physical Examination

Medical history and physical examination are very important to evaluate the severity of CD. Smoking, use of steroid, and past surgical history should be obtained; in addition, physical examination should include neurological evaluation, which should include physiological reflex, pathological reflex, and sensory and motor function test for extremities; these tests can determine the segments involved and the severity of nerve compression [27]. Furthermore, deformity check, which includes visual examination, palpation, and range of motion of the cervical spine, is very important in providing the primary information on CD [2].

### 3.2. Radiographic Evaluation

Initial radiography should be considered. Standing X-ray could show a global view of the cervical spine; in addition, extension and flexion X-ray could contribute to the determination of atlantoaxial instability and cervical flexibility. Computed tomography (CT) and magnetic resonance imaging (MRI) could help the spine surgeons determine the etiology and make a surgical plan; in addition, MRI could be performed to assess spinal cord involvement in CD [14, 32]. A three-dimensional model is an emerging investigational revolution for CD surgery. Because of the complexity of CD, it can be full of challenge to understand the location of the vertebral artery, evaluate the relationship between artery and facet of axis, and locate the altered osseous landmarks. 3D models can provide the surgeons with a clear anatomic structure [32]. Then, the clinicians can preoperatively prepare the implants with appropriate size [1]. It also contributes to the selection of the possible site for screw insertion without injuring the vertebral artery or adjacent disc space [33].

### 3.3. Scoring System

The scoring system should not be ignored in CD evaluation. The scoring system includes two kinds of assessments: HRQL assessments, which include three-level EuroQuol-5 dimensions questionnaire (EQ-5D-3L) and neck disability index (NDI), and functional assessments, which include the modified Japanese Orthopaedic Association (mJOA) questionnaire [25, 34, 35]. HRQL has several disadvantages. It was reported that although HRQL assessments were validated for general spinal diseases, they were not fully validated for CD-specific outcomes [1]. HRQL mainly contained subjective perception without radiology results [36]. In addition, the low health status in CD patient might significantly decrease the sensitivity of HRQL [29]. Therefore, several studies focused on CD patients receiving multilevel cervical fusion in the treatment of malalignment; they found that there was a significant correlation between HRQL assessments and positive sagittal balance [1, 8, 37].

## 4. Treatment Methods

Even though there is a great advancement in anesthesiology, imageology, and surgical technique, CD surgeries may still be accompanied with high complication rates, unsatisfactory clinic outcomes, and high fatality rate [14]. Several studies reported that timely surgical correction of deformity could prevent the progression of CD, while others recommended conservative therapy before considering surgery [6, 30, 38]. Therefore, the selection of treatment methods is still controversial.

### 4.1. Conservative Treatment

The primary goal of conservative therapy is to achieve pain relief and neurological recovery. The commonly used conservative treatments include physiotherapy (traction, chiropractic therapy, and cervical collar) and drug therapy (nonsteroid anti-inflammatory drugs and steroid). Compensative spinal curves usually occur in adult CD patients to reduce the rigidity of CD, while few studies have discussed how the traction will influence these compensatory curves [5]. Although few studies summarized the prognosis of CD patients who have received conservative therapies, several studies suggested that conservative therapy should be the first choice for asymptomatic congenital CD and iatrogenic CD [39, 40].

### 4.2. Surgical Treatment

Surgery should be considered in the treatment of progressive CD patients, who suffer unbearable pain or severe nerve root compression. The effect of surgical treatment is closely related to surgical approach, osteotomy, and level of fixation [41, 42]. Surgical approach determines the exposure of surgical segments and is closely related to postoperative complications [38]. Compared with posterior approaches, anterior approach is less invasive and has less complications [42]. Therefore, the anterior approach is widely used in the CD correction operations [43]. In addition, Kim et al. [42] reported that anterior osteotomy is safe and effective in treating CD. However, the anterior approach has several disadvantages: It is more difficult for the anterior approach to treat severe nerve root compression compared with the posterior approach; narrowed surgical field and restricted operable cervical segments in the anterior approach may cause malposition of the implants and then lead to postoperative deformity; the anterior approach should not be a good choice for CD correction on more than 4 segments [5, 6]. Therefore, Smith et al. [38] pointed out that only 2% of the surgeons support the application of the anterior approach in the treatment of CD.

**Figure 1:**
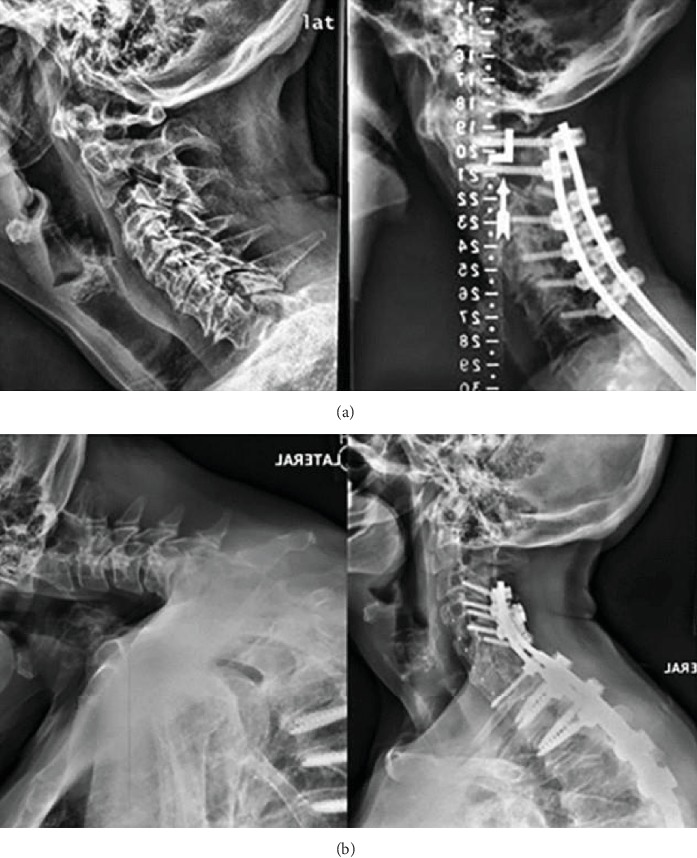
Preoperative (left) and postoperative (right) cervical lateral radiographs for (a) patient A (only improved in modified Japanese Orthopaedic Association (mJOA) scale) and (b) patient B (only improved in alignment). Baseline and 1-year cervical sagittal vertical axis (cSVA) measurements of patient A were 50.68 and 46.50 mm, respectively; in addition, his EQ-5D scores were 0.659 and 0.738, mJOA scores were 11 and 18, and neck disability index (NDI) scores were 54 and 10. For patient B, baseline and 1-year cSVA measurements were 64.44 and 49.79 mm, respectively, EQ-5D scores were 0.799 and 0.799, mJOA scores were 13 and 14, and NDI scores were 13 and 34 (cited from Passias et al. [1]).

The posterior approach is the most popular approach in the treatment of CD with a support rate of 48% [38]. However, the posterior approach should not be applied in the treatment of lordosis, because it could induce the progression of lordosis with a greater tendency of new neurological deficits (Figure 1) [31]. There is a significantly higher neurological deficit rate in the posterior approach compared with the anterior approach; however, pseudoarthrosis rate and crankshaft phenomenon rate are significantly lower in the posterior approach compared with the anterior approach [41].

**Figure 2:**
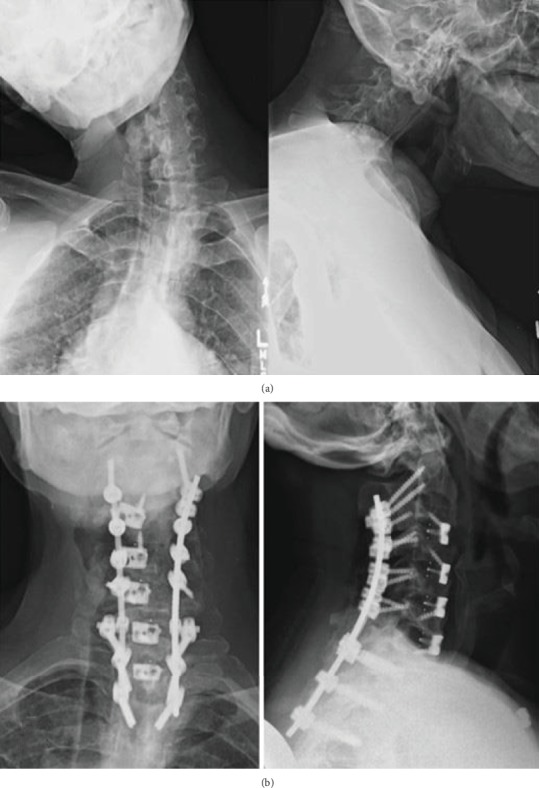
(a) CD patient's preoperative cervical X-rays demonstrating a significant coronal deformity (C2-T2 Cobb angle = 50.2°) with poor sagittal alignment (C2-7 SVA = 93.5 mm); (b) the patient's postoperative cervical X-rays demonstrating correction of fixed coronal deformity and improvement in sagittal alignment (cited from Tan and Riew [43]).

Combined posterior-anterior approach is usually recommended in the treatment of rigid CD (Figure 2) [43]. Anterior-posterior-anterior operation could provide a complete hemivertebra resection and a strong correction in the treatment of CD patients with hemivertebrae [8]. Considering the posterior-anterior approach has the highest complication rates among all approaches, this surgical approach should undergo careful planning before surgery [41]. Smith et al. [38] reported that 33% surgeons preferred anterior-posterior approach in treating rigid CD while 17% surgeons would choose anterior-posterior-anterior approach or posterior-anterior-posterior approach.

Osteotomy should be applied in the treatment of CD patients with complex multisegment fusion, history of fusion surgery, or obvious cervical imbalance [31, 44]. Smith et al. [38] reported that 41% surgeons preferred pedicle subtraction osteotomy (PSO, grade 6) or vertebral column resection (VCR, grade 7) in the correction of CD; however, others would choose multiple facet release (grade 1) or Ponte osteotomies (grade 2) in the treatment of CD; the possible explanation might be that the training background and experience of surgeons were different. Kim et al. [42] reported that anterior osteotomy was safe and effective in the correction of complex CD. This indicates that osteotomy is of great significance in the treatment of CD.

Anterior-posterior-anterior procedure was firstly used by Garg et al. [45] to treat severe, rigid posttubercular cervical spine kyphosis. This strategy included three steps. Firstly, an anterior approach should be used to osteotomize the fused vertebral body mass, decompress the spinal cord ventrally, and place a temporary cage to stabilize the spine. Secondly, the posterior approach should be used to osteotomize the fused facets and decompress the cord dorsally. Thirdly, the anterior approach should be used to replace the corpectomy cage with a larger one supplemented with an anterior cervical plate. Finally, a combination of pedicle screws and lateral mass screws should be used to correct the deformity via an anterior opening and posterior closing type of osteotomy. This strategy could help to achieve acceptable correction, improve symptoms, and avoid further progression.

## 5. Complications

CD realignment surgery is associated with more neurological and vascular complications than realignment surgery in the thoracic or lumbar spine. There were significant differences in complication rates among different approaches: 27.3% in the anterior approach, 68.4% in the posterior approach, and 79.3% in the combined approach group [6, 27, 35, 39]. However, it was reported that no preoperative factors and surgical parameters were found associated with complication occurrence [38]. Smith et al. [41] stated that early complication rate after CD realignment surgery could reach 43.6%; the most common specific complication is dysphagia; in addition, the most common complication category was new neurological deficit. Previous studies pointed out that the incidence of dysphagia was significantly higher in the combined approach (24.1%) compared with the anterior approach (8.1%) and the posterior approach (2.6%); neurological complications seemed to be more likely to occur in the posterior approach [1, 5, 7, 22, 30]. The mortality of CD correction was high. It was reported that the mortality of CD realignment surgery was 9.2%; in addition, 30-day and 90-day all-cause mortalities were 0.8% and 1.7%, respectively; however, there was no significant correlation between causes of death and surgical treatments [6, 12, 35, 46]. Therefore, the decision of surgery, in the treatment of patients with multiple preoperative complications, should be very careful. In addition, multidisciplinary consultation should be considered in the treatment of complex complications.

Understanding the natural biomechanics of the spine is very important for surgeons to make surgical strategies. Correction surgery may create new instability in the spine when it is realigned. The instability tends to cause a shift in cervical alignment; in addition, it will need stronger constant contraction to maintain the head in an upright position, which will create an extra load to cervical muscle; furthermore, as the vertebral body and disc are being wedged, the progressive malalignment might cause myelopathy [30]. Surgeons need careful planning of the surgery procedure and proper choice of internal fixation to prevent postoperative cervical instability.

## 6. Conclusions

The deficient number of cases combined with heterogeneity and multiplicity of symptoms makes it very difficult to conduct a large-sample study discussing CD. On the other hand, without a comprehensive classification system, conclusions of studies cannot be effectively unified [36]. Furthermore, most CD patients do not have obvious symptoms, which will limit the statistical analysis of CD in general population. Difficulties in the treatment of CD are obvious. Firstly, the lack of standard classification system can lead to an absence of clinical guidance. The diagnosis, evaluation, and treatment of CD patients mainly rely on surgeon's experience, experts' opinions, and a limited number of case reports. The absence of clinical evidence makes clinical determinations full of risks. Secondly, the osseous landmarks and vascular distributions can be variable in CD patients, which may cause the risk of vascular or neurological complications. A 3D model can be quite helpful in making clinical determinations. Thirdly, multiple deformities are usually presented in CD patients, which may cause chain reaction after the correction of CD. This will prevent surgeons from choosing realignment surgery that is effective but risky. In addition, the poor overall health status of CD patients can increase their postoperative mortality, which needs multidisciplinary treatments to this problem. Therefore, subsequent studies should focus on the establishment of a comprehensive symptom evaluation scale. Furthermore, multicenter clinical studies are required to investigate effects of different surgical procedures as well as associated postoperative complications.

## Figures and Tables

**Table 1:** Summary of CD classification.

Pathological features		
Congenital CD	Hypoplasia	Congenital wedge vertebra
		Congenital hemivertebra
	Segmental barriers	Unilateral massive vertebra
		Bilateral lump vertebrae
		Hybrid
	Neurofibromatosis	
Secondary CD		
Nonnoiatrogenic CD	Rheumatic disease	
	Traumatic CD	
	Metabolic diseases	
Iatrogenic CD	Operation	
	Infection	

Notice: CD: cervical deformity.

## Abbreviations

CD: Cervical deformity
CT: Computed tomography
EQ-5D-3L: EuroQuol-5 dimensions questionnaire
KFS: Klippel-Feil syndrome
mJOA: Modified Japanese Orthopaedic Association
MRI: Magnetic resonance imaging
NDI: Neck disability index
NF-1: Neurofibromatosis type 1
NF-2: Neurofibromatosis type 2
OAV: Oculoauricular vertebral
RA: Rheumatoid arthritis.
